# How leader humor stimulates subordinate boundary-spanning behavior: A social information processing theory perspective

**DOI:** 10.3389/fpsyg.2022.956387

**Published:** 2022-09-15

**Authors:** Xi Wang, Songbo Liu, Wen Feng

**Affiliations:** School of Labor and Human Resources, Renmin University of China, Beijing, China

**Keywords:** psychological safety, interpersonal influence, social information processing theory, leader humor, boundary-spanning behavior

## Abstract

Based on social information processing theory, we provide a novel theoretical account of how and when leader humor influences subordinate boundary-spanning behavior. We develop a moderated mediation model explicating the mechanism of psychological safety and the boundary condition of subordinate interpersonal influence. Using multiwave data, we tested our research hypotheses with a sample of 452 members from 140 teams in a Chinese information technology (IT) company. Results showed that leader humor positively affects subordinate boundary-spanning behavior *via* increased psychological safety. Moreover, this mediated effect is stronger when subordinates have high interpersonal influence. These findings offer theoretical and practical insights into boundary-spanning activities and leader humor, which we discuss.

## Introduction

As organizations worldwide increasingly adopt a flatter organizational structure and network-based design, team members are expected to cooperate with both internal and external stakeholders (Choi, 2002). To do so, they must cross their teams’ and even organizations’ boundaries (Ancona et al., 2002; Marrone, 2010). Boundary-spanning behavior, defined as subordinate behavior that is intended to establish relationships and interactions with external actors and helps meet team objectives (Ancona and Caldwell, 1992; Marrone et al., 2007), has thus become a common organizational phenomenon. For example, subordinates may solicit resources from higher-level leaders (Marrone et al., 2022), acquire key information from external parties (Ancona, 1990), or coordinate tasks with other teams (Ancona and Caldwell, 1992). Supporting the aforementioned examples, it is suggested that boundary-spanning behavior can protect organizations from outside threats (Aldrich and Herker, 1976), benefit organizational innovation (Hargadon, 1998), and subsequently improve organizational effectiveness (Carlisle, 2004). Effective boundary-spanning behavior is also beneficial to team learning, performance, and innovation (Hargadon, 2002; Marrone et al., 2007; Somech and Khalaili, 2014). Hence, as a promising phenomenon within the complex and dynamic work environment, boundary-spanning behavior has garnered extensive attention from scholars (e.g., Marrone et al., 2007, 2018; Marrone, 2010; Mell et al., 2022).

Traditionally, managing team boundary is the responsibility of formal leaders (e.g., Morgeson et al., 2010; Marrone et al., 2018). Due to the increasing need for extensive collaboration both within and across teams, however, team members are also expected to proactively engage in boundary-spanning activities (e.g., Marrone et al., 2007; Marrone, 2010). According to Marrone’s (2010) multilevel review of the team boundary-spanning literature, “team boundary-spanning actions originate from the behaviors and actions of its individual members” (p. 913). Therefore, it is valuable to conduct research on boundary-spanning behavior at the individual level. Prior work has elucidated that subordinates carrying out boundary-spanning behavior can improve their status, influence, performance, and innovation through gaining unique information and opportunities (Hargadon, 2002; Tortoriello et al., 2014; Liu et al., 2016; Shah et al., 2018); however, they can also experience role overload in meeting contradictory expectations among the internal and external parties (Kahn et al., 1964; Katz and Kahn, 1978; Marrone et al., 2007). Further, when leaders view subordinate boundary-spanning behavior as weakening their control, they may attempt to undermine such boundary spanners (Mell et al., 2022). Due to this dual nature of boundary spanning, it is important to examine determinants that may shape the tendency of subordinate boundary-spanning behavior.

Researchers have demonstrated that leaders play a key role in subordinate boundary-spanning activities (Joshi et al., 2009; Marrone, 2010). For example, leaders could motivate subordinates to engage in boundary-spanning behavior through strategic propositions and supportive coaching (Ancona, 1990; Marrone et al., 2022). Although these existing studies have made significant contributions toward our understanding of how leaders direct, encourage, and support subordinate boundary-spanning behavior, they underscore that the potential interpersonal risks for boundary spanners may come from the leaders themselves. That is, subordinates who step up to exhibit boundary-spanning behavior may encounter the formal leaders’ undermining due to weakening their control over the team (Mell et al., 2022). Despite the common portrait of boundary-spanning behavior as a worthy, needed, and encouraged endeavor (Morgeson et al., 2010), subordinates sometimes see risks for themselves if they step up to do so (Zhang et al., 2020; Lee Cunningham et al., 2022). Consequently, more research is urgently needed on how leaders can help alleviate subordinates’ concerns on the potential interpersonal risks involved in boundary activities and motivate them to proactively carry out boundary-spanning behavior.

Accordingly, this study aims to answer the above research question through a social information processing theory perspective, which posits that subordinates actively seek to understand and behave according to the norms and expectations in their organizations by processing social information cues (Salancik and Pfeffer, 1978). Serving as a key information source, leaders’ behaviors play a vital role in shaping subordinates’ perceptions of their work environment (Shamir et al., 1993) and guiding their attitudes and behaviors (Chiu et al., 2016). Research has found that leader humor—the extent to which leaders use humor to interact with subordinates—could provide positive social information cues (Yam et al., 2018) that reduce hierarchical distance between leaders and subordinates (Graham, 1995; Mesmer-Magnus et al., 2012) and help alleviate subordinates’ concerns about potential risks at work (Cooper et al., 2018). Therefore, we propose that leader humor could increase subordinates’ willingness to take interpersonal risks (i.e., psychological safety) (Edmondson, 1999) and engage in boundary-spanning behavior.

Notwithstanding the above arguments, the effect of leader humor on subordinate boundary-spanning behavior may vary among subordinates due to their different abilities in sensing the social information cues and calibrating their behavior, as captured by the concept of interpersonal influence (Ferris et al., 2005). Interpersonal influence enables subordinates to capture the social information cues of leader humor and adapt their attitude and behavior accordingly, thus strengthening the effect of leader humor on subordinate boundary-spanning behavior *via* psychological safety. Hence, we propose that subordinates with high interpersonal influence are more likely to respond to leader humor and develop psychological safety, which subsequently facilitates boundary-spanning behavior.

**FIGURE 1:**
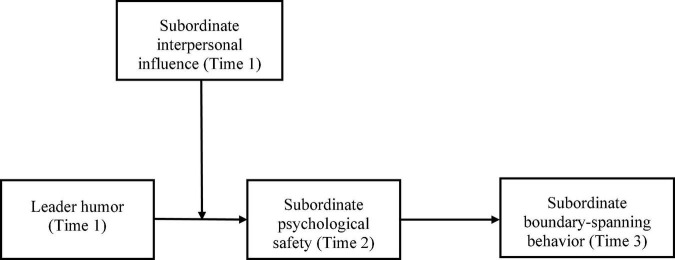
Theoretical model.

We tested our theoretical model by using a sample of 452 subordinates from 140 teams in a Chinese information technology (IT) company, which provided supportive results. Our research makes three main contributions. First, we enrich the theoretical understandings of leadership factors on subordinate boundary-spanning behavior by identifying the role of leader humor. Second, we uncover the mediating role of psychological safety in linking leader humor to subordinate boundary-spanning behavior. Third, we propose subordinate interpersonal influence as a boundary condition that explicates the diverse subordinate responses to leader humor, transferring our insights to consider the question of why leader humor has different effects on subordinate boundary-spanning behavior. Our theoretical model is demonstrated in Figure 1.

## Theory and hypotheses development

### Leader humor and subordinate boundary-spanning behavior

The use of humor by leaders with their subordinates is receiving growing scholarly interest (e.g., Cooper et al., 2018; Yam et al., 2018; Tan et al., 2020; Zhang and Su, 2020). To date, there have been two dominant perspectives on the definition of leader humor, namely, viewing it as a trait or as a behavior. From the trait perspective, Martin and Lefcourt (1984) considered humor to be an individual difference in abilities or tendencies to create and perceive humorous stimuli. Martin et al. (2003) categorized humor into four styles (i.e., affiliative, aggressive, self-enhancing, and self-defeating humor), and Martin (2001) defined leader sense of humor as a leader’s tendency to display amusing behaviors and attitudes in social interactions. Alternatively, from the behavioral perspective, Cooper (2005) defined humor as an intentional and social communication, while Cooper et al. (2018) used leader humor to refer to the extent to which leaders express humor toward their subordinates.

In this research, we are interested in humor as a behavior rather than sense of humor as a trait (Martin and Lefcourt, 1984), as this definition is broad enough to include all types and forms of humor (e.g., affiliative humor, visual images) (Cooper, 2008) and also captures interpersonal phenomenon through actor sharing and target perceiving (Cooper, 2005), which is appropriate in our research. In line with prior work (Pundt and Herrmann, 2015; Kim et al., 2016; Cooper et al., 2018; Carnevale et al., 2022), we consider leader humor at the individual level because members of the same work group may have different perceptions of their leader’s humor. Past research has illustrated the positive effects of leader humor on job satisfaction, organizational citizenship behavior, and team performance (Hughes and Avey, 2009; Mao et al., 2017; Cooper et al., 2018). According to social information processing theory (Salancik and Pfeffer, 1978), subordinates attend to social cues passed on through leader humor and exhibit positive responses (Yam et al., 2018). Psychological safety captures a psychological state, which refers to whether a subordinate feels safe taking interpersonal risks (Edmondson, 1999). In this respect, we expect that when leaders express humor at work, subordinate psychological safety is more likely to be advanced, for several reasons.

Leader humor reduces hierarchical distance between leaders and subordinates in organizations (Graham, 1995; Mesmer-Magnus et al., 2012), which implicitly symbolizes leaders’ willingness to violate hierarchical systems and deemphasize the hierarchy (Cooper, 2008). Moreover, leader humor displays leaders’ inclination to show vulnerability and to have more uncomplicated and open exchanges with subordinates (Yam et al., 2018). When subordinates perceive leader humor, they are more likely to receive leaders’ relationship-oriented signals with friendliness and supportiveness (Blau, 1964; Cooper, 2008), thus fostering mutual trust and support in interpersonal interactions. Subordinates also rely on the information from leader humor to make sense of the environment (Shamir et al., 1993), and have greater potential to feel secure in interpersonal risk-taking (Detert and Edmondson, 2011). All of these outcomes may contribute to a stronger sense of subordinate psychological safety. Hence, our first hypothesis is as follows:

*Hypothesis 1*. Leader humor is positively related to subordinate psychological safety.

Boundary-spanning behavior refers to subordinate efforts to establish relationships with external parties to achieve team tasks and objectives (Ancona and Caldwell, 1992). Although boundary-spanning can bring benefits to subordinates (Hargadon, 2002; Tortoriello et al., 2014; Liu et al., 2016; Shah et al., 2018), it can also be challenging and risky for subordinates to engage in boundary-spanning behavior (Marrone, 2010). Boundary spanners might encounter inconsistent expectations from the internal and external entities (Katz and Kahn, 1978; Choi, 2002). Moreover, because subordinate boundary activities weaken the leader’s territorial control over the team, they could even result in leaders’ undermining (Mell et al., 2022). Given the potential risks for boundary spanners, we expect subordinate psychological safety to be a necessary antecedent of subsequent boundary-spanning behavior. When subordinates perceive psychological safety by sensing the positive social information cues of leader humor, they are more likely to access high levels of trust and mutual respect (Kahn, 1990), and thus feel safe to establish relationships and interact with external parties without fear of negative consequences (Marrone et al., 2007).

Combining the first hypothesis with the above arguments, we propose that psychological safety works as a mechanism that links leader humor and subordinate boundary-spanning behavior. In line with social information processing theory (Salancik and Pfeffer, 1978), subordinates who sense the social information cues of leader humor will have positive responses and enhanced psychological safety, increasing the likelihood that they will take an active part in boundary-spanning activities. On the basis of these arguments, we predict as follows:

*Hypothesis 2*. Psychological safety mediates the relationship between leader humor and subordinate boundary-spanning behavior.

### The moderating role of subordinate interpersonal influence

To understand when leader humor brings more positive consequences, we again follow social information processing theory (Salancik and Pfeffer, 1978) and propose that subordinates’ relational skills may influence the process of addressing social information. Interpersonal influence reflects subordinates’ ability to adapt and calibrate their behavior to different situations (Ferris et al., 2005). Subordinates who maintain high levels of interpersonal influence are capable of utilizing their observations and keen understanding to adjust their behavior to situations (Ferris et al., 2005). Interpersonal influence is associated with the potential effects of leader humor because subordinates who possess high levels of interpersonal influence tend to calibrate their behavior to leader humor, which stimulates subsequent development of psychological safety. We expect subordinate interpersonal influence is key to solve the puzzle of when leader humor contributes to more positive effects.

From the social information processing theory perspective, subordinates with different levels of interpersonal influence will attend to the signals that leaders pass on through humor during interpersonal interactions and react accordingly with their own responses (Salancik and Pfeffer, 1978). As noted, when subordinates have high levels of interpersonal influence, they capitalize on their observations and appropriately adapt their behavior to the situation (Ferris et al., 2005). Faced with declining hierarchical distance as signaled through leader humor (Graham, 1995; Mesmer-Magnus et al., 2012), subordinates with high interpersonal influence tend to make sense of leader humor and tailor their initiatives (Wihler et al., 2017). Such subordinates are thus more likely to react with positive responses (Gervais and Wilson, 2005; Lynch, 2010), thereby increasing psychological safety.

In stark contrast, subordinates who possess low levels of interpersonal influence are less sensitive to social cues and also less capable of calibrating their behaviors to the situation (Ferris et al., 2005). When leaders interact in humorous ways (Yam et al., 2018), subordinates with low interpersonal influence may not interpret the social information cues of leader humor as deemphasizing the hierarchical difference, so they will not adjust their attitudes and behaviors (Ferris et al., 2005, 2007). As such, these subordinates may not properly calibrate and present social responses to leader humor (Wihler et al., 2017), leading to decreased psychological safety. Accordingly, we hypothesize the following:

*Hypothesis 3*. Subordinate interpersonal influence moderates the relationship between leader humor and subordinate psychological safety, such that the relationship is stronger when subordinate interpersonal influence is high.

Building on the above hypotheses, we further propose a pattern of moderated mediation, in which subordinate interpersonal influence moderates the indirect relationship between leader humor and subordinate boundary-spanning behavior *via* psychological safety. The strength of this indirect relationship is determined by the level of subordinate interpersonal influence. Specifically, when subordinates have high levels of interpersonal influence, they are more likely to feel psychologically safe through processing the information of leader humor, thereby engaging in more boundary-spanning behavior. Thus, we propose the following hypothesis:

*Hypothesis 4*: Subordinate interpersonal influence moderates the indirect effect between leader humor and subordinate boundary-spanning behavior *via* psychological safety, such that the indirect effect is stronger when subordinate interpersonal influence is high.

## Materials and methods

### Sample and procedures

To test our hypotheses, we turned to a large division of an IT company located in Beijing as the host for our research. With the approval and assistance of the company’s human resources directors, we contacted subordinates nested in teams *via* email and asked them to participate in a voluntary research study involving three stages of data collection with a 2-week time lag. The subordinates were engineers and technicians who worked collaboratively in work teams to set up and maintain IT systems for corporate clients, which made them an appropriate sample for our research. The time lag ensured minimized disruption for the subordinates (on the basis of discussions with management) and allowed the research team to manage the concerns about survey length and common method bias (Podsakoff et al., 2003). In order to alleviate evaluation apprehension and encourage candidness, we assured participants of the confidentiality of their responses. At Time 1, we invited 576 randomly selected subordinates to participate in our study. Among them, 550 subordinates returned surveys that provided information on perceived leader humor, interpersonal influence, and demographic data. At Time 2 (2 weeks later), 512 subordinates completed a survey rating their psychological safety. At Time 3 (2 weeks after Time 2), 479 subordinates provided ratings on their own boundary-spanning behavior.

After data cleaning and matching the three-stage responses, we had final responses from 452 subordinates in 140 teams, constituting a final effective response rate of 78%. Among the 452 subordinates, 73% were male and 51% were aged 31–40. In terms of education, 95% of the subordinates had a bachelor’s degree or above. Moreover, 78% reported that they had worked with their supervisors for more than 5 years.

### Measures

The three surveys were translated from English into Mandarin Chinese using a double-blind back-translation procedure (Brislin, 1986). All measures used were mature scales evaluated on a six-point Likert-type scale ranging from 1 (strongly disagree) to 6 (strongly agree) unless otherwise specified.

#### Leader humor

We measured leader humor with a three-item scale developed by Cooper et al. (2018). Subordinates were asked to evaluate the extent to which their leaders displayed humor. A sample item is “My supervisor jokes around me.” Cronbach’s alpha was 0.76 in this study.

#### Interpersonal influence

We measured interpersonal influence with a four-item scale from the Political Skill Inventory (PSI) developed by Ferris et al. (2005). The prior work on interpersonal influence shows that it is appropriate to use one of the dimensions of the PSI that fits with the focus of one’s research (e.g., Brockner et al., 2004; Thompson, 2005; Ng and Feldman, 2010; Baer, 2012). Subordinates were asked to evaluate the extent of their interpersonal influence. A sample item is “I am able to communicate easily and effectively with others.” Cronbach’s alpha was 0.85 in this study.

#### Psychological safety

We measured psychological safety with a seven-item scale developed by Edmondson (1999). Subordinates were asked to evaluate the level of their psychological safety. A sample item is “It is safe to take a risk on this team.” Cronbach’s alpha was 0.86 in this study.

#### Boundary-spanning behavior

We measured boundary-spanning behavior with a six-item scale developed by Marrone et al. (2007). Subordinates were asked to evaluate the extent to which they engage in boundary-spanning behavior. A sample item is “I can persuade outsiders (e.g., faculty, clients) to support our team decisions.” Cronbach’s alpha was 0.78 in this study.

#### Control variables

Following existing studies (e.g., Cooper, 2005; Pundt, 2015; Goswami et al., 2016; Hussain and Shahzad, 2019), we selected the subordinate’s gender (0 = male; 1 = female), age, and education (1 = secondary school or below; 2 = junior college; 3 = bachelor; 4 = master; 5 = doctorate) and the duration of the supervisor-subordinate relationship (i.e., dyadic tenure) as the main control variables of the hypothesized relationships among leader humor, subordinate psychological safety, subordinate boundary-spanning behavior, and subordinate interpersonal influence.

### Analytical strategies

Given that our data had a nested structure with several subordinates nested in the same teams, it was necessary to address the interdependence issue (Bliese et al., 2018). We therefore used Mplus 7.0 to analyze our data through multilevel path analyses. Leader humor, psychological safety, boundary-spanning behavior, and interpersonal influence were all set as Level 1 variables that should be group-mean centered. We adopted Monte Carlo bootstrapping to construct 95% confidence intervals (CIs), evaluating the significance of the indirect effects in our model (Preacher and Selig, 2012).

## Results

### Confirmatory factor analyses

We examined the hypothesized measurement model with four factors (leader humor, interpersonal influence, psychological safety, and boundary-spanning behavior) and conducted a series of confirmatory factor analyses to establish the discriminant validity of our measurement model. The fit statistics of the hypothesized four-factor model indicated an acceptable fit: χ^2^ (164) = 329.28, CFI = 0.95, TLI = 0.94; RMSEA = 0.05; SRMR = 0.05. This four-factor model was significantly better than a three-factor model in which interpersonal influence and leader humor were combined into one factor [χ^2^ (167) = 610.54, CFI = 0.86, TLI = 0.84; RMSEA = 0.08; SRMR = 0.07]; a two-factor model in which interpersonal influence, leader humor, and psychological safety were combined into one factor [χ^2^ (169) = 1366.05, CFI = 0.63, TLI = 0.58; RMSEA = 0.13; SRMR = 0.12]; and a single-factor model in which interpersonal influence, leader humor, psychological safety, and boundary-spanning behavior were combined into one factor [χ^2^ (170) = 1575.25, CFI = 0.56, TLI = 0.51; RMSEA = 0.14; SRMR = 0.12]. The above findings supported the discriminant validity of our research variables.

### Common method bias

Although we adopted a multiwave design, being faced with the problem of common method bias was still a possibility, we utilized Harman’s single-factor test to examine the possible issue (Podsakoff et al., 2003). Based on the results of Harman’s single-factor test, we found that the largest factor accounted for 29.70% (less than 40%) of the variance; this was also consistent with the aforementioned results of the confirmatory factor analyses, which demonstrated that the single-factor model did not fit the data well. Therefore, common method bias was not a serious problem and was unlikely to confound the interpretation of our results.

### Hypotheses tests

Table 1 shows descriptive statistics and correlations among our variables. Table 2 presents the multilevel path analytic results. Leader humor was significantly and positively associated with subordinate psychological safety (*B* = 0.13, *p* < 0.05), supporting Hypothesis 1. Further, psychological safety was significantly and positively related to subordinate boundary-spanning behavior (*B* = 0.35, *p* < 0.01). We also examined the unstandardized indirect effect coefficients relevant to Hypothesis 2 by using the Monte Carlo simulation to construct the CIs. Based on 5,000 re-samples, the results showed that psychological safety had a significant mediation effect on the relationship between leader humor and subordinate boundary-spanning behavior (indirect effect = 0.05, 95% CI = [0.01, 0.09], excluding zero). Thus, Hypothesis 2 was supported.

**TABLE 1:** Descriptive statistics and correlations.

Variables	*M*	*SD*	1	2	3	4	5	6	7	8
1. Subordinate gender	0.27	0.44	−							
2. Subordinate age	2.63	0.70	−0.10*	−						
3. Subordinate education	3.24	0.53	0.10*	–0.04	−					
4. Dyadic tenure	3.46	1.12	−0.15**	0.72**	−0.31**	−				
5. Leader humor	4.26	0.82	0.06	0.03	0.07	–0.03	(0.76)			
6. Subordinate interpersonal influence	4.57	0.71	0.03	0.03	0.02	–0.01	0.36**	(0.85)		
7. Subordinate psychological safety	4.85	0.60	–0.02	−0.10*	0.08	−0.10*	0.21**	0.24**	(0.86)	
8. Subordinate boundary-spanning behavior	3.85	0.56	0.01	0.05	–0.02	0.02	0.38**	0.36**	0.47**	(0.78)

*N* = 452 subordinates. Reliability of variables is listed in parentheses. *p < 0.05, **p < 0.01.

**TABLE 2:** Multilevel path analytic results of hypotheses.

Variables	Psychological safety		Boundary-spanning behavior
***Control variables***			
Subordinate gender	−0.15 (0.08)	−0.13 (0.08)	0.03 (0.06)
Subordinate age	−0.07 (0.07)	−0.06 (0.08)	0.10 (0.07)
Subordinate education	0.12 (0.08)	0.09 (0.07)	−0.05 (0.06)
Dyadic tenure	0.02 (0.05)	0.02 (0.05)	0.00 (0.04)
***Independent variable***			
Leader humor	0.13* (0.05)	0.09 (0.06)	0.17** (0.04)
*Moderator*			
Subordinate interpersonal influence		0.22** (0.04)	
***Interaction term***			
Leader humor × Subordinate interpersonal influence		0.17** (0.05)	
***Mediator***			
Psychological safety			0.35** (0.06)
Pseudo *R*^2^	0.07	0.12	0.15

*N* = 452 subordinates in 140 teams. Unstandardized regression coefficients are reported. Pseudo *R*^2^ indicates the proportional reduction in the total variance of variables. *p < 0.05, **p < 0.01.

**FIGURE 2:**
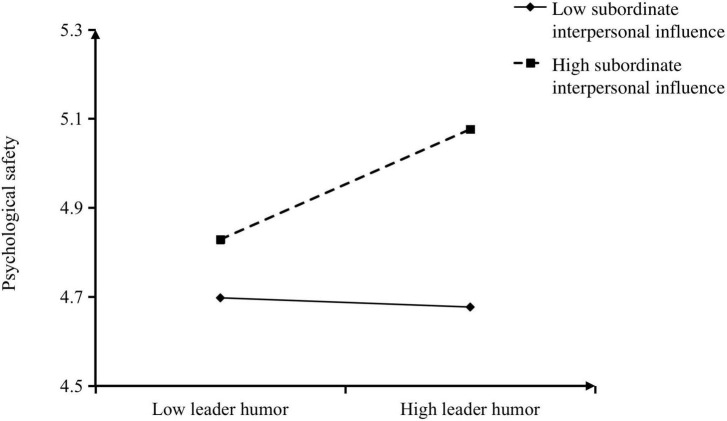
The moderating role of subordinate interpersonal influence on the relationship between leader humor and subordinate psychological safety.

As shown in Table 2, we found a positive interaction effect between leader humor and subordinate interpersonal influence in predicting psychological safety (*B* = 0.17, *p* < 0.01). Figure 2 shows an interaction plot based on values ± 1 standard deviation from the mean of the moderating variable (i.e., subordinate interpersonal influence; Cohen et al., 2003). The results of the simple slope analysis showed that the relationship between leader humor and psychological safety was significantly positive when subordinate interpersonal influence was high (simple slope = 0.19, *p* < 0.01), but insignificant when it was low (simple slope = −0.02, *n.s.*). Thus, Hypothesis 3 was also supported.

Finally, we followed a Monte Carlo-based re-sampling approach to test the model in an integrated fashion. The indirect effect of leader humor on subordinate boundary-spanning behavior *via* psychological safety was significantly positive when subordinate interpersonal influence was high (conditional indirect effect = 0.04, 95% CI = [0.01, 0.08], excluding zero), but insignificant when subordinate interpersonal influence was low (conditional indirect effect = 0.004, 95% CI = [−0.04, 0.05], including zero). Overall, the above empirical evidence supported Hypothesis 4’s prediction that subordinate interpersonal influence moderated the indirect effect of leader humor on subordinate boundary-spanning behavior *via* psychological safety, such that the indirect effect was stronger when subordinate interpersonal influence was high.

## Discussion

Based on social information processing theory, our results yield two nuanced insights regarding how and when leader humor drives subordinate boundary-spanning behavior. First, leader humor improves subordinate psychological safety, which in turn increases their engagement in boundary-spanning behavior. Second, the process linking leader humor and subordinate boundary-spanning behavior *via* psychological safety is amplified when subordinates have high levels of interpersonal influence. Overall, our research findings extend the literature in several valuable ways and also generate certain practical implications.

### Theoretical implications

Our research makes multiple theoretical extensions and has a number of implications for the existing literature. First, our work fills a gap in the literature on boundary-spanning behavior by identifying leader humor as one of the determinants of subordinate boundary-spanning behavior from a social information processing perspective. Given that leaders are of great importance in fostering boundary-spanning activities (Mell et al., 2022), considerable attention has been given to their potential role in stimulating subordinate boundary-spanning behavior. For example, leaders’ supportive coaching behaviors provide a necessary support to help subordinates engage in boundary-spanning behavior (Marrone et al., 2022), and charismatic and transformational leaders also endorse subordinate boundary-spanning activities (Knipfer et al., 2018; Wadei et al., 2021). However, prior work has not underscored the potential risks to subordinates from carrying out boundary-spanning behavior, such as role overload (Kahn et al., 1964; Katz and Kahn, 1978) and even leaders’ undermining (Mell et al., 2022). Our research thus complements this work by providing insight into how the psychological state that leader humor fosters can reduce subordinates’ negative concerns through positive social cues; this ensures that subordinates feel safe, which stimulates their boundary-spanning behavior.

Second, our research sheds light on an essential mechanism of psychological safety and integrates the humor literature and boundary-spanning literature at a level of detail that has previously been overlooked in both strands of literature. Scholars have primarily focused on the value of boundary-spanning self-efficacy (Marrone et al., 2022) and perspective taking (Wadei et al., 2021) in explaining the significance of leaders for boundary spanning according to its positive points. Our research complements this existing work by capturing how challenging and taxing boundary-spanning behavior can be for subordinates (Marrone, 2010) and highlighting the role of psychological safety to explain the positive function of leader humor in addressing these negative points. Identifying the underlying psychological mechanism also responds to calls for greater theoretical understanding of the determinants of subordinates’ boundary-spanning behavior as noted above (Joshi et al., 2009; Marrone, 2010). In this way, our research underscores the mechanism of psychological safety in linking leader humor and subordinates’ boundary-spanning behavior, providing nuanced and valuable insights into such behavior.

Finally, our study lends credence to the unique boundary condition of subordinate interpersonal influence, which explains different reactions to leader humor from different subordinates. According to the literature review by Marrone (2010), although team members are actually on behalf of their teams to achieve boundary-spanning activities, the amount and type of their contributions may vary (Chan, 1998); this suggests that it is necessary to explore boundary-spanning behavior at the individual level. Our results on the moderating role of subordinate interpersonal influence add to the limited knowledge on the boundary conditions of leader humor’s effects. Therefore, considering subordinate interpersonal influence as a key contingency facilitates a more comprehensive understanding of these effects on subordinate boundary-spanning behavior.

### Practical implications

Our findings also have the following practical implications. First, our work explores how leader humor stimulates subordinate boundary-spanning behavior, such that when leaders express humor in interactions, subordinates are more likely to have positive responses and engage in boundary-spanning behavior. To this end, we recommend that leaders display humor in social interactions, thereby contributing to positive effects on subordinate psychological state and behavior. Additionally, organizations should strive to integrate humor into leadership training courses (Goswami et al., 2016) and encourage leaders to cultivate and adopt humor.

Second, our research illustrates the mechanism of psychological safety in the relationship between leader humor and subordinate boundary-spanning behavior, which strengthens the argument that subordinate boundary-spanning behavior is driven by the psychological safety established by leader humor. Organizations can thus provide some supportive practices for subordinates to develop and improve their psychological safety, such as mentoring (Newman et al., 2017). At the same time, it is critical that supervisors guide subordinates to develop high levels of psychological safety (Frazier et al., 2017) and put them in positions that require boundary-spanning activities.

Third, our research shows the moderating role of subordinate interpersonal influence and explicates that subordinates with high interpersonal influence are prone to feel psychological safety and enact boundary-spanning behavior when faced with leader humor. Accordingly, organizations should include interpersonal influence as a selection criterion in the job interview process and provide training programs for subordinates to elevate their awareness of leader behavior (Deng et al., 2016). Moreover, subordinates can seize the opportunities to develop and improve their interpersonal influence in mentoring activities, which will help them better understand leader behavior and take appropriate actions.

### Limitations and directions for future research

Primary among the limitations of this research is our research design. Although we adopted a multiwave design to reduce potential common method bias (Podsakoff et al., 2003), this design did not allow us to draw absolute causal conclusion. It is possible that the extent to which subordinates engage in boundary-spanning behavior may develop their level of psychological safety (Faraj and Yan, 2009). Future research should adopt longitudinal or experimental designs to further justify the strong causal relationship between leader humor and subordinate boundary-spanning behavior.

In addition, in this research, we have generally conceptualized leader humor as a social behavior (Cooper et al., 2018) and offered a theoretical account for the link between leader humor and subordinate boundary-spanning behavior. Martin et al. (2003) categorized humor into four specific types, including affiliative, agressive, self-enhancing and self-defeating humor. Therefore, exploring the effects of different styles of leader humor on subordinate psychological safety and subsequent boundary-spanning behavior may offer an important avenue for future research.

Notably, we captured how leader humor influences subordinate boundary-spanning behavior from a social information processing perspective and uncovered the mediating mechanism of this relationship. Yet we only considered psychological safety as a mediator; there may be other mechanisms (e.g., affective processes) that explain the connection between leader humor and subordinate boundary-spanning behavior. Accordingly, it will be necessary to explore other underlying mechanisms in the future to enrich our current results.

Finally, we examined how leader humor can influence subordinate boundary-spanning behavior at the individual level, in line with social information processing theory (Salancik and Pfeffer, 1978). However, leader humor is also considered to be a team-level phenomenon (Tremblay, 2017), such that it may help teams develop shared psychological safety and foster team-level boundary-spanning behavior. To date, there has been little research on the effects of leader humor from this team perspective. Hence, consistent with the suggestions from Wei et al. (2022), we encourage future research to explore the impacts of leader humor at the team level as well.

## Conclusion

From the standpoint of social information processing theory, we theoretically and empirically investigate how and when leader humor sparks subordinate boundary-spanning behavior. Our research results highlight that leader humor plays an important role in stimulating subordinate psychological safety and further boundary-spanning behavior, which is strengthened by subordinate interpersonal influence. We hope that our research provides future directions in the boundary-spanning behavior literature and offers managers practical insights into managing boundary-spanning activities in organizations.

## Data availability statement

The raw data supporting the conclusions of this article will be made available by the authors, without undue reservation.

## Author contributions

XW was involved in conceptualizing the research, data analysis, and writing – original manuscript. SL was responsible for the data collection and the manuscript revision. WF was responsible for the theory building, data analysis, and manuscript revision. All authors contributed to the research and approved the submission of this version.

## References

[B1] Aldrich H. Herker D. (1976). Boundary spanning roles and organizational structure. *Acad. Manag. J.* 2 217–230. 10.5465/amr.1977.4409044

[B2] Ancona D. G. (1990). Outward bound: Strategies for team survival in an organization. *Acad. Manag.* 33 334–365. 10.2307/256328

[B3] Ancona D. G. Caldwell D. F. (1992). Bridging the boundary: External activity and performance in organizational teams. *Adm. Sci. Q.* 37 634–665. 10.2307/2393475

[B4] Ancona D. Bresman H. Kaeufer K. (2002). The comparative advantage of X-teams. *MIT Sloan Manag. Rev.* 43 33–40. 10.1287/mksc.21.2.209.151 19642375

[B5] Baer M. (2012). Putting creativity to work: The implementation of creative ideas in organizations. *Acad. Manag. J.* 55 1102–1119. 10.5465/amj.2009.0470

[B6] Blau P. M. (1964). *Exchange and power in social life.* New York, NY: John Wiley.

[B7] Bliese P. D. Maltarich M. A. Hendricks J. L. (2018). Back to basics with mixed-effects models: Nine take-away points. *J. Bus. Psychol.* 33 1–23. 10.1007/s10869-017-9491-z

[B8] Brislin R. W. (1986). “The wording and translation of research instruments,” in *Field methods in cross-cultural research*, eds Lonner W. J. Berry J. W. (Beverly Hills, CA: Sage), 137–164.

[B9] Brockner J. Spreitzer G. Mishra A. Hochwarter W. Pepper L. Weinberg J. (2004). Perceived control as an antidote to the negative effects of layoffs on survivors’ organizational commitment and job performance. *Adm. Sci. Q.* 49 76–100. 10.2307/4131456

[B10] Carlisle P. R. (2004). Transferring, translating and transforming: An integrative framework for managing knowledge across boundaries. *Org. Sci.* 15 555–568. 10.1287/orsc.1040.0094 19642375

[B11] Carnevale J. B. Huang L. Yam K. C. Wang L. (2022). Laughing with me or laughing at me? The differential effects of leader humor expressions on follower status and influence at work. *J. Org. Behav.* 43, 1153–1171. 10.1002/job.2629

[B12] Chan D. (1998). Functional relations among constructs in the same content domain at different levels of analysis: A typology of composition models. *J. Appl. Psychol.* 83 234–246. 10.1037/0021-9010.83.2.234

[B13] Chiu C. Y. C. Owens B. P. Tesluk P. E. (2016). Initiating and utilizing shared leadership in teams: The role of leader humility, team proactive personality, and team performance capability. *J. Appl. Psychol.* 101 1705–1720. 10.1037/apl0000159 27618409

[B14] Choi J. N. (2002). External activities and team effectiveness. *Small Group Res.* 33 181–208. 10.1177/104649640203300202

[B15] Cohen J. Cohen P. West S. G. Aiken L. S. (2003). *Applied multiple regression/correlation analysis for the behavioral sciences*, 3rd Edn. Mahwah, NJ: Lawrence Erlbaum.

[B16] Cooper C. D. (2005). Just joking around? Employee humor expression as an ingratiatory behavior. *Acad. Manag. Rev.* 30 765–776. 10.5465/amr.2005.18378877

[B17] Cooper C. D. (2008). Elucidating the bonds of workplace humor: A relational process model. *Hum. Relat.* 61 1087–1115. 10.1177/0018726708094861

[B18] Cooper C. D. Kong D. T. Crossley C. D. (2018). Leader humor as an interpersonal resource: Integrating three theoretical perspectives. *Acad. Manag. J.* 61 769–796. 10.5465/amj.2014.0358

[B19] Deng H. Guan Y. Wu C. H. Erdogan B. Bauer T. Yao X. (2016). A relational model of perceived over-qualification: The moderating role of interpersonal influence on social acceptance. *J. Manag.* 44 1–23. 10.1177/0149206316668237

[B20] Detert J. R. Edmondson A. C. (2011). Implicit voice theories: Taken-for-granted rules of self-censorship at work. *Acad. Manag. J.* 54 461–488. 10.5465/AMJ.2011.61967925

[B21] Edmondson A. C. (1999). Psychological safety and learning behavior in work teams. *Adm. Sci. Q.* 44 350–383. 10.2307/2666999

[B22] Faraj S. Yan A. (2009). Boundary work in knowledge teams. *J. Appl. Psychol.* 94 604–617. 10.1037/a0014367 19450002

[B23] Ferris G. R. Treadway D. C. Kolodinsky R. W. Hochwarter W. A. Kacmar C. J. Douglas C. (2005). Development and validation of the political skill inventory. *J. Manag.* 31 126–152. 10.1177/0149206304271386

[B24] Ferris G. R. Treadway D. C. Perrewé P. L. Brouer R. L. Douglas C. Lux S. (2007). Political skill in organizations. *J. Manag.* 33 290–320. 10.1177/0149206307300813

[B25] Frazier M. L. Fainshmidt S. Klinger R. L. Pezeshkan A. Vracheva V. (2017). Psychological safety: A meta-analytic review and extension. *Pers. Psychol.* 70 113–165. 10.1111/peps.12183

[B26] Gervais M. Wilson D. S. (2005). The evolution and functions of laughter and humor: A synthetic approach. *Q. Rev. Biol.* 80 395–430. 10.1086/498281 16519138

[B27] Goswami A. Nair P. Beehr T. Grossenbacher M. (2016). The relationship of leaders’ humor and employees’ work engagement mediated by positive emotions. *Leadersh. Org. Dev. J.* 37 1083–1099. 10.1108/lodj-01-2015-0001

[B28] Graham E. E. (1995). The involvement of sense of humor in the development of social relationships. *Commun. Rep.* 8 158–169. 10.1080/08934219509367622

[B29] Hargadon A. B. (1998). Firms as knowledge brokers: Lessons in pursing continuous innovation. *Calif. Manag. Rev.* 40 209–227. 10.2307/41165951

[B30] Hargadon A. B. (2002). Brokering knowledge: Linking learning and innovation. *Res. Org. Behav.* 24 41–85. 10.1016/S0191-3085(02)24003-4

[B31] Hughes L. W. Avey J. B. (2009). Transforming with levity: Humor, leadership, and follower attitudes. *Leadersh. Org. Dev. J.* 30 540–562. 10.1108/01437730910981926

[B32] Hussain S. Shahzad K. (2019). The effects of supervisor organizational embodiment and organizational identification on the LMX-creativity relationship. *South Asian J. Manag. Sci.* 13 99–115. 10.21621/sajms.2019132.01

[B33] Joshi A. Pandey N. Han G. (2009). Bracketing team boundary spanning: An examination of task-based, team-level, and contextual antecedents. *J. Org. Behav.* 30 731–759. 10.1002/job.567

[B34] Kahn R. L. Wolfe D. M. Quinn R. P. Snoek J. D. Rosenthal R. A. (1964). *Organizational stress: Studies in role conflict and ambiguity.* New York, NY: Wiley.

[B35] Kahn W. A. (1990). Psychological conditions of personal engagement and disengagement at work. *Acad. Manag. J.* 33 692–724. 10.2307/256287

[B36] Katz D. Kahn R. L. (1978). *The social psychology of organizations.* New York, NY: Wiley.

[B37] Kim T. Y. Lee D. R. Wong N. Y. S. (2016). Supervisor humor and employee outcomes: The role of social distance and affective trust in supervisor. *J. Bus. Psychol.* 31 125–139. 10.1007/s10869-015-9406-9

[B38] Knipfer K. Schreiner E. Schmid E. Peus C. (2018). The performance of pre-founding entrepreneurial teams: The importance of learning and leadership. *Appl. Psychol. Int. Rev.* 67 401–427. 10.1111/apps.12126

[B39] Lee Cunningham J. Sonday L. Ashford S. J. (2022). Do I Dare? The psychodynamics of anticipated image risk, leader identity endorsement, and leader emergence. *Acad. Manag. J.* 10.5465/amj.2018.1258 [Epub ahead of print].

[B40] Liu S. Jiang K. Chen J. Pan J. Lin X. (2016). Linking employee boundary spanning behavior to task performance: The influence of informal leader emergence and group power distance. *Int. J. Hum. Resour. Manag.* 29 1879–1899. 10.1080/09585192.2016.1216872

[B41] Lynch O. (2010). Cooking with humor: In-group humor as social organization. *Humor* 23 127–159. 10.1515/humr.2010.007

[B42] Mao J. Y. Chiang T. J. Zhang Y. Gao M. (2017). Humor as a relationship lubricant: The implications of leader humor on transformational leadership perceptions and team performance. *J. Leadersh. Org. Stud.* 24 494–506. 10.1177/1548051817707518

[B43] Marrone J. A. (2010). Team boundary spanning: A multilevel review of past research and proposals for the future. *J. Manag.* 36 911–940. 10.1177/0149206309353945

[B44] Marrone J. A. Ferraro H. S. Huston T. (2018). A theoretical approach to female team leaders’ boundary work choices. *Group Org. Manag.* 43 1–32. 10.1177/0159601118795384

[B45] Marrone J. A. Quigley N. R. Prussia G. E. Dienhart J. (2022). Can supportive coaching behaviors facilitate boundary spanning and raise job satisfaction? An indirect-effects model. *J. Manag.* 48 1131–1159. 10.1177/01492063211003951

[B46] Marrone J. A. Tesluk P. E. Carson B. A. (2007). A multilevel investigation of antecedents and consequences of team member boundary spanning behavior. *Acad. Manag. J.* 50 1423–1439. 10.2307/20159482

[B47] Martin R. A. (2001). Humor, laughter, and physical health: Methodological issues and research findings. *Psychol. Bull.* 127 504–519. 10.1037/0033-2909.127.4.504 11439709

[B48] Martin R. A. Lefcourt H. M. (1984). Situational humor response questionnaire: Quantitative measure of sense of humor. *J. Pers. Soc. Psychol.* 47 145–155. 10.1037/0022-3514.47.1.145

[B49] Martin R. A. Puhlik-Doris P. Larsen G. Gray J. Weir K. (2003). Individual differences in uses of humor and their relation to psychological well-being: Development of the humor styles questionnaire. *J. Res. Pers.* 37 48–75. 10.1016/S0092-6566(02)00534-2

[B50] Mell J. N. Quintane E. Hirst G. Carnegie A. (2022). Protecting their turf: When and why supervisors undermine employee boundary spanning. *J. Appl. Psychol.* 107 1009–1019. 10.1037/apl0000960 34766797

[B51] Mesmer-Magnus J. Glew D. J. Viswesvaran C. (2012). A meta-analysis of positive humor in the workplace. *J. Manag. Psychol.* 27 155–190. 10.1108/02683941211199554

[B52] Morgeson F. P. Derue D. S. Karam E. P. (2010). Leadership in teams: A functional approach to understanding leadership structures and processes. *J. Manag.* 26 5–39. 10.1177/0149206309347376

[B53] Newman A. Donohue R. Eva N. (2017). Psychological safety: A systematic review of the literature. *Hum. Resour. Manag. Rev.* 27 521–535. 10.1016/j.hrmr.2017.01.001

[B54] Ng T. W. Feldman D. C. (2010). The effects of organizational embeddedness on development of social capital and human capital. *J. Appl. Psychol.* 95 696–712. 10.1037/a0019150 20604589

[B55] Podsakoff P. M. MacKenzie S. B. Lee J. Y. Podsakoff N. P. (2003). Common method biases in behavioral research: A critical review of the literature and recommended remedies. *J. Appl. Psychol.* 88 879–903. 10.1037/0021-9010.88.5.879 14516251

[B56] Preacher K. J. Selig J. P. (2012). Advantages of Monte Carlo confidence intervals for indirect effects. *Commun. Methods Meas.* 6 77–98. 10.1080/19312458.2012.679848

[B57] Pundt A. (2015). The relationship between humorous leadership and innovative behavior. *J. Manag. Psychol.* 30 878–893. 10.1108/jmp-03-2013-0082

[B58] Pundt A. Herrmann F. (2015). Affiliative and aggressive humour in leadership and their relationship to leader-member exchange. *J. Occup. Org. Psychol.* 88 108–125. 10.1111/joop.12081

[B59] Salancik G. R. Pfeffer J. (1978). A social information processing approach to job attitudes and task design. *Adm. Sci. Q.* 23 224–253. 10.2307/2392563 10307892

[B60] Shah N. P. Levin D. Z. Cross R. (2018). Secondhand social capital: Boundary spanning, secondhand closure, and individual performance. *Soc. Networks* 52 18–27. 10.1016/j.socnet.2017.04.005

[B61] Shamir B. House R. J. Arthur M. B. (1993). The motivational effects of charismatic leadership: A self-concept based theory. *Org. Sci.* 4 577–594. 10.1287/orsc.4.4.577 19642375

[B62] Somech A. Khalaili A. (2014). Team boundary activity: Its mediating role in the relationship between structural conditions and team innovation. *Group Org. Manag.* 39 274–299. 10.1177/1059601114525437

[B63] Tan L. Wang Y. Qian W. Lu H. (2020). Leader humor and employee job crafting: The role of employee-perceived organizational support and work engagement. *Front. Psychol.* 11:499849. 10.3389/fpsyg.2020.499849 33117214 PMC7578259

[B64] Thompson J. A. (2005). Proactive personality and job performance: A social capital perspective. *J. Appl. Psychol.* 90 1011–1017. 10.1037/0021-9010.90.5.1011 16162073

[B65] Tortoriello M. McEvily B. Krackhardt D. (2014). Being a catalyst of innovation: The role of knowledge diversity and network closure. *Org. Sci.* 26 423–438. 10.1287/orsc.2014.0942 19642375

[B66] Tremblay M. (2017). Humor in teams: Multilevel relationships between humor climate, inclusion, trust, and citizenship behaviors. *J. Bus. Psychol.* 32 363–378. 10.1007/s10869-016-9445-x

[B67] Wadei K. A. Lu C. Wu W. (2021). Unpacking the chain mediation process between transformational leadership and knowledge worker creative performance: Evidence from China. *Chin. Manag. Stud.* 15 483–498. 10.1108/cms-03-2020-0118

[B68] Wei H. Shan D. Wang L. Zhu S. (2022). Research on the mechanism of leader aggressive humor on employee silence: A conditional process model. *J. Vocat. Behav.* 135 1–12. 10.1016/j.jvb.2022.103717

[B69] Wihler A. Blickle G. Ellen B. P. Hochwarter W. A. Ferris G. R. (2017). Personal initiative and job performance evaluations: Role of political skill in opportunity recognition and capitalization. *J. Manag.* 43 1388–1420. 10.1177/0149206314552451

[B70] Yam K. C. Christian M. S. Wei W. Liao Z. Y. Nai J. (2018). The mixed blessing of leader sense of humor: Examining costs and benefits. *Acad. Manag. J.* 61 348–369. 10.5465/amj.2015.1088

[B71] Zhang C. Nahrgang J. D. Ashford S. J. Derue D. S. (2020). The risky side of leadership: Conceptualizing risk perceptions in formal leadership and investigating the effects of their overtime changes in teams. *Org. Sci.* 31 1138–1158. 10.1287/orsc.2019.1350 19642375

[B72] Zhang J. J. Su W. L. (2020). Linking leader humor to employee innovative behavior: The roles of work engagement and supervisor’s organizational embodiment. *Front. Psychol.* 11:592999. 10.3389/fpsyg.2020.592999 33381068 PMC7767835

